# Supplementary material for: Principles for coding associative memories in a compact neural network
Source: eLife. 2023 May 4;12:e74434. doi: 10.7554/eLife.74434 (PMC10159626; doi:10.7554/eLife.74434)
Supplement: Transparent reporting form [file elife-74434-transrepform1.pdf]

Westbrook Centre
Milton Road
Cambridge CB4 1YG, UK

P 01223 855340
W elifesciences.org
T @elife

eLife's transparent reporting form

We encourage authors to provide detailed information *within their submission* to facilitate the interpretation and replication of experiments. Authors can upload supporting documentation to indicate the use of appropriate reporting guidelines for health-related research (see EQUATOR Network), life science research (see the BioSharing Information Resource), or the ARRIVE guidelines for reporting work involving animal research. Where applicable, authors should refer to any relevant reporting standards documents in this form.

If you have any questions, please consult our Journal Policies and/or contact us: editorial@elifesciences.org.

Sample-size estimation

- You should state whether an appropriate sample size was computed when the study was being designed
- You should state the statistical method of sample size computation and any required assumptions
- If no explicit power analysis was used, you should describe how you decided what sample (replicate) size (number) to use

Please outline where this information can be found within the submission (e.g., sections or figure legends), or explain why this information doesn't apply to your submission:

We could not compute a-priori, at the design stage of the study, the appropriate sample size. We embarked into a high-throughput analysis of as many neurons as possible to reveal those that participate in memory formation and storage. We ended up analyzing dynamics of multiple neurons from nearly 300 animals, far more than any previous report.
This comprehensive analysis included all four types of associative memories and a careful design to include all necessary controls (Naïve, trained, and mock-trained controls for each learning paradigms).

Replicates

- You should report how often each experiment was performed
- You should include a definition of biological versus technical replication
- The data obtained should be provided and sufficient information should be provided to indicate the number of independent biological and/or technical replicates
- If you encountered any outliers, you should describe how these were handled
- Criteria for exclusion/inclusion of data should be clearly stated
- High-throughput sequence data should be uploaded before submission, with a private link for reviewers provided (these are available from both GEO and ArrayExpress)

Please outline where this information can be found within the submission (e.g., sections or figure legends), or explain why this information doesn't apply to your submission:

eLife Sciences Publications, Ltd is a limited liability non-profit non-stock corporation incorporated in the State of Delaware, USA, with company number 5030732, and is registered in the UK with company number FC030576 and branch number BR015634 at the address Westbrook Centre, Milton Road, Cambridge CB4 1YG.

1) Behavioral experiments:

Repeats:

Choice behavior - Figure 1, Figure 1-figure supplement 1&2, and Figure 4-figure supplement 5:

In these assays, we quantified the choice behavior of a population of animals.
A technical repeat is a single choice test - one experimental plate. A biological repeat is the average of 3 such technical repeats that were performed on the same day. Biological repeats were carried out on different days using independent synchronized worm cultures (following bleaching). This is because most of the variance was observed between experimental days. The variance between technical repeats, performed within the same day, was rather low and largely affected by variations in the experimental setup (positioning of endpoints, etc.).

Biological repeats were typically performed 4-8 times. Naïve animals were tested up to 21 times since they were included in each of the different assayed groups.
Each of the four training paradigms is tested against a paradigm-specific mock-trained group and a common naive group.
Location in the manuscript: Information about repeats is denoted in the figure legend as well as in the bar graphs themselves where individual biological repeats are evident as dots.

Quantifying locomotion parameters, Figures 8:

In these analyses, in each experimental repeat we tracked ~100 animals at a time resulting in thousands of tracks. Experiments for each group were replicated on 4-21 different days. Due to the employed analysis, data from within groups was pooled to weight the contribution of each animal equally.

Location in the manuscript: Information on how many experimental repeats (days) are denoted in the figure legend. Dots in the scatter plots denote biological repeats.

Data exclusion:

Choice behavior Fig 1, Figure 1-figure supplement 2:

The day-to-day variance in choice behavior was high. Choice behavior is quantified by the choice index that ranges from -1 to 1. The learning index is then calculated by subtracting the choice index of the control group from the choice index of the choice index of trained groups. Due to presumably environmental changes, choice behavior can depart from the usual mean choice index creating ceiling effects. This often coincided with environmental changes such as construction work in the building or seasonal weather changes (i.e., temperature shifts). In appetitive regimes, the choice indices of naive, mock-trained and trained groups would be 1, making the detection of positive increase in the choice index impossible because of saturation of the measurement scale. Therefore, the following elimination rule was implemented for appetitive training regimes: If choice indices of all three groups was higher than 0.8, data was discarded (the normal naive mean is around 0).

eLife Sciences Publications, Ltd is a limited liability non-profit non-stock corporation incorporated in the State of Delaware, USA, with company number 5030732, and is registered in the UK with company number FC030576 and branch number BR015634 at the address Westbrook Centre, Milton Road, Cambridge CB4 1YG.

Location in the manuscript: This outlined in the section 'Statistical analysis' of the Material and Methods part.

2) Calcium imaging:

Repeats:

Calcium imaging Figures 2, 3, 4, and 5

Responses of individual neurons were collected. Individual worms are biological repeats. For each condition, we typically assayed ~10 animals, with up to 8 trials per worm. These trials were averaged to get one response per animal and neuron that was then used for statistics. Imaging duration was limited due to signal bleaching and possible physiological effects.

Location in the manuscript: Animal numbers are stated in the figure legends throughout the main text and the suppl. figures. These numbers are also evident from the heat maps and the dots in bar graphs.

Data exclusion:

Measurements from animals were discarded if animals showed no residual movement during imaging (even levamisole-paralyzed animals show residual motion).

Outliers

Given the high variability in the neural activities, only measurements with fluorescent activity within a 95% confidence interval were included in the statistics.

Location in the manuscript: This is outlined in the section 'Statistical analysis' of the Material and Methods.

Statistical reporting

- Statistical analysis methods should be described and justified
- Raw data should be presented in figures whenever informative to do so (typically when N per group is less than 10)
- For each experiment, you should identify the statistical tests used, exact values of N, definitions of center, methods of multiple test correction, and dispersion and precision measures (e.g., mean, median, SD, SEM, confidence intervals; and, for the major substantive results, a measure of effect size (e.g., Pearson's r, Cohen's d)
- Report exact p-values wherever possible alongside the summary statistics and 95% confidence intervals. These should be reported for all key questions and not only when the p-value is less than 0.05.

eLife Sciences Publications, Ltd is a limited liability non-profit non-stock corporation incorporated in the State of Delaware, USA, with company number 5030732, and is registered in the UK with company number FC030576 and branch number BR015634 at the address Westbrook Centre, Milton Road, Cambridge CB4 1YG.

Please outline where this information can be found within the submission (e.g., sections or figure legends), or explain why this information doesn't apply to your submission:

1) Behavior

Statistical methods

Choice tests figure 1:
To eliminate day-to-day variance in choice of butanone that is not associated with learning and memory, statistical tests were carried out based upon the learning index **(LI),** and not the choice indices **(CI)** of the individual groups. Since the LI is the delta between CIs of the same day, this eliminates day-to-day variations in butanone choice. Learning indices were then tested against 0 using one-sample t-tests (paired t-test) and adjusted for multiple comparisons (see below, Statistical metrics, multiple comparisons). Experience-dependent modulation of choice behavior (=Learning) occurred when the LI was significantly different from 0.
Location in the manuscript: This is discussed in detail in suppl. figure-figure supplement 4 and stated in Figure 1 caption.

Locomotion behavior Figure 8.
Differences between all groups were detected using an ANOVA on ranks. Differences in locomotion parameters between individual groups were detected by pairwise comparisons based on Wilcoxon rank-sum test/signed rank test because some groups did not appear to follow normal distribution (see Figure 8D-H) and did not pass normality tests. Pairwise comparisons adjusted for multiple comparisons (see below, Statistical metrics, multiple comparisons).
Location in the manuscript: Material and Methods section (general explanation) and Figure legends (state individual tests, used for pairwise comparisons).

Raw data presentation

Raw data is presented throughout all behavioral figures of the manuscript.

Statistical metrics

In all figures depicting behavioral data, the data center is denoted by the mean, except for the scatter plots in Figures 8 D,F,H. In those plots, both mean (thick black line) and median (thinner black line) are used to provide a better estimation of data centers and distributions. The SEM was used as the metric of precision in all line plots and bar plots with error indicators. Raw data were always presented alongside the metrics for center and precision identifying sample size and providing a better estimation of the underlying distribution.

p-Values were adjusted for multiple comparisons by positive False Discovery Rate (Storey 2002). Comparison design for pairwise comparisons is listed in Table 1 in the section 'statistical analysis' in the Material and Methods part.
Location in the manuscript: Material and Methods section.

eLife Sciences Publications, Ltd is a limited liability non-profit non-stock corporation incorporated in the State of Delaware, USA, with company number 5030732, and is registered in the UK with company number FC030576 and branch number BR015634 at the address Westbrook Centre, Milton Road, Cambridge CB4 1YG.

p-Values

The manuscript involves many experimental groups, and consequently, many more comparisons. We explicitly provide all the significant values. To avoid text overload, we did not mention the non-significant measures

.

Location in the manuscript: p-Values are denoted in all the figures in which testing occurred In all figures depicting behavioral data, the data center is denoted by the mean. The SEM was used as the metric of precision in all line plots and bar plots with error indicators. Raw data are always presented alongside the metrics for center and precision identifying sample size and providing a better estimation of the underlying distributions.

eLife Sciences Publications, Ltd is a limited liability non-profit non-stock corporation incorporated in the State of Delaware, USA, with company number 5030732, and is registered in the UK with company number FC030576 and branch number BR015634 at the address Westbrook Centre, Milton Road, Cambridge CB4 1YG.

2) Imaging neural activity

Statistical methods

Differences between all groups were detected using an ANOVA. Differences in the neural activation after stimulus exchange between individual experimental groups were detected by pairwise comparisons based on t-test and then adjusted for multiple comparisons (see below, Statistical metrics, multiple comparisons).
Neural activities of all neurons were classified into the underlying training conditions using k means-nearest-neighbor, random forest, and neural net algorithms. A portion of the data was allocated to training while the classification accuracy was measured on a held-out portion of the dataset as the macro F1 score.
Multivariate regression models were run on neural activities of AIY neurons and sensory neurons. AIY activities were regressed on different combinations of sensory activities. Regression models were evaluated by Cross-validation and F-statistics. Differences in neural activations between memory conditions were subjected to a Principal component analysis.
Location in the manuscript: Material and Methods section (general explanation) and in each figure legend.

Raw data presentation

Raw data are presented throughout all activity imaging figures of the manuscript. Raw activity graphs are presented in heat maps. Integrated intensity metrics that form the basis for statistical comparisons are presented as scatter dots in the bar graphs.
Location in the manuscript: In all neuronal activity figures.

Statistical metrics

In all figures depicting activity data, the data center is denoted by the mean. The SEM was used as the metric of precision in all line plots with error indicators. Raw data (biological repeats) are always presented in scatter plots alongside the metrics for center and precision, identifying sample size and providing a better estimation of the underlying distributions. We state the F1 scores as metric for classification accuracy in Figure 4 and Figure 4-figure supplement 7. For the regression analyses in Figure 6 and Figure 6-figure supplement 1, we state the regression coefficients with the according p-values alongside the R^2 of the overall model and average R^2 from cross-validation based on the held-out dataset.

eLife Sciences Publications, Ltd is a limited liability non-profit non-stock corporation incorporated in the State of Delaware, USA, with company number 5030732, and is registered in the UK with company number FC030576 and branch number BR015634 at the address Westbrook Centre, Milton Road, Cambridge CB4 1YG.

p-Values

The manuscript involves many experimental groups, and consequently, many more comparisons. We explicitly provide all the significant values. To avoid text overload, we did not mention the non-significant measures.

Location in the manuscript: p-Values are denoted in all the figures.

(For large datasets, or papers with a very large number of statistical tests, you may upload a single table file with tests, Ns, etc., with reference to sections in the manuscript.)

Group allocation

- Indicate how samples were allocated into experimental groups (in the case of clinical studies, please specify allocation to treatment method); if randomization was used, please also state if restricted randomization was applied
- Indicate if masking was used during group allocation, data collection and/or data analysis

Please outline where this information can be found within the submission (e.g., sections or figure legends), or explain why this information doesn't apply to your submission:

All data acquisition and analysis was automated using computer software, so no masking was needed. Calcium dynamics imaging was collected using a confocal automated system and image analyses were performed using developed code. Worm counting and locomotion parameters were carried out using a camera and software with no manual intervention.

Additional data files ("source data")

- We encourage you to upload relevant additional data files, such as numerical data that are represented as a graph in a figure, or as a summary table
- Where provided, these should be in the most useful format, and they can be uploaded as "Source data" files linked to a main figure or table
- Include model definition files including the full list of parameters used
- Include code used for data analysis (e.g., R, MatLab)
- Avoid stating that data files are "available upon request"

Please indicate the figures or tables for which source data files have been provided:

Data availability:

All the data concerning behavior and activity responses of individual neurons, are already fully available through the OSF public repository domain:
https://osf.io/5v4qu/

Code availability

All the code that was used to generate each of the figures is available at https://osf.io/5v4qu/ and in:
https://github.com/ChristianPritz/Principles-for-coding-associative-memories-in-a-compact-neural-network

eLife Sciences Publications, Ltd is a limited liability non-profit non-stock corporation incorporated in the State of Delaware, USA, with company number 5030732, and is registered in the UK with company number FC030576 and branch number BR015634 at the address Westbrook Centre, Milton Road, Cambridge CB4 1YG.
